# A Generative Adversarial Network Based a Rolling Bearing Data Generation Method Towards Fault Diagnosis

**DOI:** 10.1155/2022/7592258

**Published:** 2022-07-13

**Authors:** Lin Huo, Huanchao Qi, Simiao Fei, Cong Guan, Ji Li

**Affiliations:** ^1^Shenyang Aerospace University, Shenyang 110035, China; ^2^Liaoning Key Laboratory of Aircraft Safety and Airworthiness, Shenyang, China; ^3^Shenyang Aircraft Design Institute, Shenyang 110035, China; ^4^Nanjing University, Nanjing 210023, China

## Abstract

As a new generative model, the generative adversarial network (GAN) has great potential in the accuracy and efficiency of generating pseudoreal data. Nowadays, bearing fault diagnosis based on machine learning usually needs sufficient data. If enough near-real data can be generated in the case of insufficient samples in the actual operating condition, the effect of fault diagnosis will be greatly improved. In this study, a new rolling bearing data generation method based on the generative adversarial network (GAN) is proposed, which can be trained adversarially and jointly via a learned embedding, and applied to solve fault diagnosis problems with insufficient data. By analyzing the time-domain characteristics of rolling bearing life cycle monitoring data in actual working conditions, the operation data are divided into three periods, and the construction and training of the generative adversarial network model are carried out. Data generated by adversarial are compared with the real data in the time domain and frequency domain, respectively, and the similarity between the generated data and the real data is verified.

## 1. Introduction

Nowadays, as an important mechanical component, rolling bearings have an indispensable position in mechanical rotating equipment. 30% of the failures of mechanical rotating equipment are due to the failure of rolling bearings, so the fault diagnosis of rolling bearings is extremely important. Most traditional fault detection methods for rolling bearings are based on human judgment and data-driven algorithms with existing knowledge and theories [1–6]. In the diagnosis, the focus is on the numerical calculation of effective characteristic parameters or the extraction of signals, and the fault is often analyzed and diagnosed in the established digital set. Therefore, human subjective factors will have a certain impact on the analysis results and reduce the accuracy of the analysis results [7–9]. At the same time, for safety and economic considerations, most of the bearings are in safe working state during operation, and most of the collected data are working data in a healthy state, but the working data under fault conditions are extremely lacking. This also makes it difficult to apply data-driven algorithms normally, and the results obtained lack practical reference value. In modern times, with the continuous development of deep learning and artificial neural network technologies, advanced machine learning algorithms have been widely used in the field of fault diagnosis. When these deep learning methods deal with the problem of class imbalance, due to their bias towards most classes [10–12], their accuracy will also be reduced. As an emerging generative model, the generative adversarial network method uses two different neural networks to confront each other and has the ability of projecting the standard data distribution to the high-dimensional real-world data distribution to generate high-accuracy pseudoreal data that makes it a place in the field of fault diagnosis and prognosis.

Fuan et al. [13] proposed an adaptive deep convolutional neural network for rolling bearing fault diagnosis, which reduces the dependence on manual experience to a certain extent by automatically learning the essential fault features layer by layer from the input data. Guo et al. [14] used the Hilbert envelope spectrum and support vector machine to analyze the amplitude modulated pulse in the vibration signal of rolling bearing, and accurately diagnosed many kinds of faults of rolling bearing. Shao et al. [15] used an optimized deep belief network and applied it to the analysis of rolling bearing simulation signals and experimental signals. These research results show high accuracy and feasibility, but there are also some certain shortcomings. No matter what kind of neural network model is used, the final result is always inseparable from the simplification of the vibration signal, such as feature reduction by setting a threshold, and simplifying the vibration signal to a single signal; these methods have a certain theoretical basis, but in actual working conditions, however, certain differences inevitably exist. Nowadays, the neural network theory has spawned many branches, and the practical problems applied to data fusion have gradually increased. However, in the fault diagnosis field, the application of neural networks has always been restricted. It is impossible to simulate the complex situation of the real world no matter what kind of traditional generative model has been used. This kind of huge life cycle data will have some problems for the fitting model. Traditional simulation data generation often involves methods such as MLE (maximum likelihood estimation), Markov chain methods, and approximation methods [16], which are subject to complex calculations, the pros and cons of simulation are affected by other factors, and gradient disappearance. As a result, the data generation efficiency is low and the accuracy is low.

In this environment, GAN (generative adversarial networks) came into being [17]. Through the joint action of the two modules of the generator and the discriminator, it can effectively solve the existing low accuracy problems of data generated by deep learning and neural network models while generating a large amount of simulation data. Some large artificial intelligence company such as Google, OpenAI, and Facebook are using this feature to explore kinds of GAN applications [18]. However, the application of GAN has mainly focused on the generation and processing of images [19], which has not been widely used in the field of fault diagnosis. In recent years, some scholars have begun to think about the application value of GAN as a generative model in the field of fault diagnosis. Gao et al. [20] used GAN to generate a large number of fault samples and applied them to fault diagnosis based on FEM simulation and reflected the practicability of GAN from the side through the simulation results of different classifiers, and achieved certain results. Hua et al. [21] proposed a GAN-based fault diagnosis method for imbalanced data. At the same time, new GAN networks such as WGAN [22], CGAN, and BiGAN have also been proposed successively, making the application of GAN in the field of fault diagnosis a reality. However, some of these studies are limited to the structural innovation of GAN to further improve the performance of GAN and are not applied to actual working conditions; although, some are applied to actual working conditions, the demonstration focuses on the results of fault diagnosis, not the accuracy of GAN itself. Properties and application value. Further applications of GANs in the field of fault diagnosis and the advancement of GANs compared to other generative models remain to be studied.

This paper mainly discusses the bearing data game generation method based on GAN, which effectively solves the problems of fault diagnosis methods caused by insufficient data. The accuracy and application value of the data generated by GAN are fully demonstrated through the values of mean, root mean square, skewness, and other values of the rolling bearing in three different periods and a large number of image comparisons. Compared with other data simulations, GAN has great advantages. It can realize strong nonlinear data fitting function, prevent human interference, and automatically extract the fault characteristics through the computer. The data model based on GAN have higher reliability and can also be used under more extensive working conditions. Facing the development of intelligence and data informatization, it is a trend to combine artificial intelligence and deep learning with rolling bearing fault diagnosis. This combination can promote an intelligent diagnosis model and improve the accuracy and efficiency.

## 2. Related Work

In the field of rolling bearing failure research, compared with the traditional thinking “diagnosis + processing” thinking mode [23], the modern failure prognosis theory can not only prevent the occurrence of failures more effectively and reduce the consequences of accidents but can also effectively reduce maintenance costs. Most of the modern research studies on rolling bearing faults are single-point fault signal modeling [24]. Most of this method is by analyzing the vibration characteristics of a single point of rolling bearings, collecting vibration data obtained in a short period of time, and eliminating the influence of other failure modes through assumptions. This research method can analyze the single failure mode of rolling bearing in a relatively targeted manner [25], but it is difficult to analyze the multipoint compound fault and the coupling caused by it under the actual working conditions [26].

At present, many scholars have advanced and mature research methods in fault diagnosis. Zhang et al. [27] used deep adversarial learning to realize automatic identification of unknown failure modes, which has an extremely high practical application value under the condition of strong data uncertainty; Wen et al. [28] used TCNN (ResNet-50) for fault diagnosis and applied it to three different datasets, all of which have a prediction accuracy of 99%; Jiao et al. [29] used the residual joint adaptation adversarial network (RJAAN) for fault diagnosis, which can learn category discrimination and domain-invariant feature information for cross-domain fault diagnosis, which has strong robustness and superiority; Zhang et al. [30] used federated learning to solve the data island problem in fault diagnosis while ensuring the privacy of different clients, which provided the possibility for further application of confidential decentralized learning in fault diagnosis. Most of these excellent fault diagnosis methods require a large amount of rolling bearing fault data to support, and the lack of this type of data also greatly limits the application value of data-driven algorithms in actual working conditions. The acquisition of bearing fault diagnosis data is roughly divided into two types: (1) Based on real data collected by the instrument in a short time [31]. (2) Based on simulation data generated by deep learning or neural network. The first method often has special assumptions, such as approximating the vibration signal to smooth signal [32] and simplifying the failure mode of the rolling bearing to one. These assumptions often do not match the actual operating conditions. It is difficult to have higher accuracy. By the second method, it is difficult to guarantee the accuracy of the data, and the large amount of simulation data generated has a low reference value. Although there have been many research studies on rolling bearing fault diagnosis through machine learning algorithms, researchers such as Gunerkar et al. [33] have proposed an artificial neural network that uses wavelet transform as a noise reduction tool and extracts sensitive time-series parameters. Another example is convolutional neural network (CNN), convolutional sparse combination learning (CSCL), and other deep learning methods [34]. However, in the process of data input, the variables under various working conditions must be controlled artificially. Moreover, it is difficult to guarantee the application ability of the generated pseudoreal data under actual working conditions. Based on the generative adversarial network, this paper proposes a rolling bearing simulation data generation platform, which can effectively utilize the characteristics of GAN that can generate large amounts of data and generate high accuracy.

In 2014, Goodfellow proposed a new generative network model [35]. This new network model is very different from the traditional generative network. In the structure of the entire network, it not only contains a generative network but also has a discriminant network [36]. There is an antagonistic relationship between these two parts, and the idea of this antagonistic relationship is mainly derived from the game theory. In this theory, both parties are required to be equal in the game and then change their strategies according to the opponent's strategy changes, so as to achieve the goal of winning in the game. To extend and integrate this theory into the confrontation network needs the generator and the discriminator to be the two sides of the game in the game. The generator can fit the data generation to generate model samples. The optimization goal is to be able to finally achieve Nash equilibrium and finally achieve the generator's estimation and prediction of the overall distribution of sample data.

The generative model of the GAN can be used to model the distribution of real data and generate simulation data [37]. Compared with the real data, these generated data have extremely high similarity. Therefore, this model can be applied well in unsupervised learning, semi-supervised learning, and multi-learning.

Compared with the traditional rolling bearing fault diagnosis method, GAN abandons the influence of human subjective factors on the results and avoids the problem of poor applicability caused by fault data only applicable to specific fault types. The large amount of data it generates can help the fault diagnosis results based on data-driven algorithms to be closer to the actual working conditions. Also, different from traditional neural networks and deep learning methods [38], the adversarial relationship between the discriminator and the generator eliminates the need for variational lower bounds or approximate inference during data generation; it also avoids the calculation of the partition function caused by the repeated application of the Markov chain learning mechanism. As a new generation of generative models, the GAN solves the problems of low efficiency and inaccurate generated data of traditional generative models. It also has the idea of discriminative models and uses the confrontation between the generator and the discriminator to greatly improve the accuracy and speed of the generated data. At the same time, the limitation of the generation sample dimension and loss function of the traditional generative model is also solved in this new generative model, which also makes the GAN have a very high degree of model design freedom and greatly enhances the possibility of its practical application. A large number of innovations and application methods have emerged in just six years after GAN was available [39–41], which also made its application in the industrial field possible.

Combined with fault diagnosis theory, the application of GAN can effectively solve the problem of insufficient life cycle data that limits fault diagnosis. Inspired by this, we choose rolling bearings that are widely used in industry and has serious failure consequences for research, and use the GAN method to game generate rolling bearing pseudoreal data. By analyzing the time-domain characteristics of the rolling bearing life cycle monitoring data under actual working conditions, the operating data are divided into three periods to construct and train the generative adversarial network model. The adversarial generated data are compared with the real data in the time domain and frequency domain, respectively, to verify the similarity between the generated data and the real data. The actual bearing life cycle data are from the University of Cincinnati. The GAN model is used to generate the rolling bearing data and then verify the similarity of generated data and real data in the time domain and frequency domain.

## 3. Generative Adversarial Network Method

### 3.1. Generative Adversarial Network

**Figure 1:**
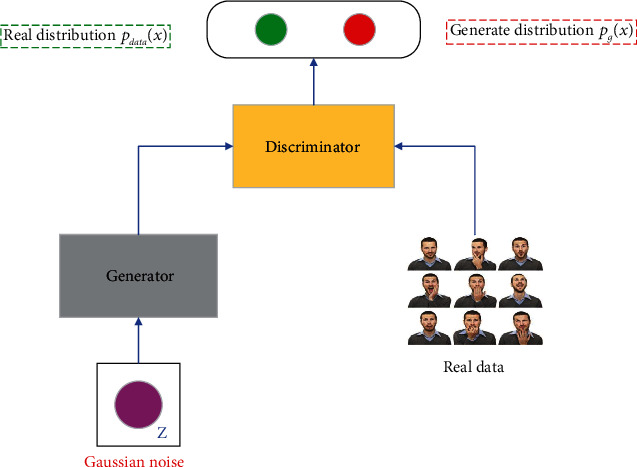
Generative adversarial network principle.

The generation method is an important branch of the machine learning method. It involves the learning of distribution assumptions and distribution parameters of explicit or implicit variables of the data, and sampling new samples based on the learned model. The principle of the generative model is shown in Figure 1; each point represents an image, sampled from the real data distribution *P*_data_(*x*), and the real data distribution area represents real image data. A Gaussian noise distribution *P*_*g*_(*x*) is input into the generation model, so that the output of the generation model is as close as possible to the real data distribution *P*_data_(*x*) so as to accurately approximate the real data. For the choice of objective function, traditional generative models often use the maximum likelihood function as the objective function. However, the GAN introduces a discriminant model in addition to the generative model and achieves the purpose of optimization by adversarial training of the generative model and the discriminant model.

**Figure 2:**
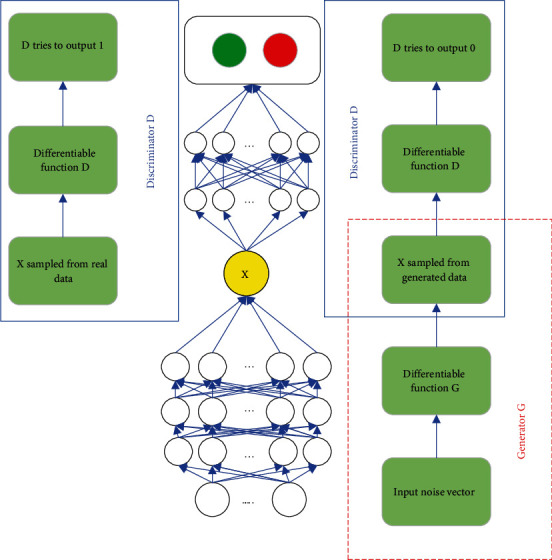
GAN process framework.

Under the guidance of the binary zero-sum game, the framework of the GAN also contains a pair of opposite models, namely, discriminator and generator. The discriminator is mainly used to make reasonable and correct judgments and distinctions between real data and the generated data by the generator and improve the accuracy of network identification data. The role of the generator is to ensure that the generated data are as close to the actual distribution as possible within a limited range. Therefore, in order to win in the game, both sides need to continuously improve their discrimination and generation ability, so as to achieve the goal of optimizing the entire power generation network, and finally find the Nash equilibrium between the two. The specific GAN framework is shown in Figure 2. It can be seen that the input of the generator is a random noise vector from the public probability distribution. The output is pseudoreal data generated by the computer. The input of the discriminator is the picture *x*, which can be sampled. The output of the discriminator is scalar, which is used to represent the probability that *x* is real data. In other words, when the discriminator considers *x* to be real data, its output is 1, otherwise, it is 0. The discriminator and generator are optimized repeatedly. When the discriminator cannot accurately distinguish the data source, it can be considered that the generator has learned the distribution of real data samples.

### 3.2. Objective Function

The objective function means that the generator and discriminator in the GAN can be any differentiable function, which needs to be optimized by the stochastic gradient descent method SGD. The first condition of using the SGD method is to establish an objective function that can judge and supervise the learning effect. When the generator *G* is given, the optimization of the discriminator *D* is the same as the training effect of the conventional binary classifier, so the objective function can be expressed by the cross entropy, which is as follows:

(1) $$\begin{array}{c} J\left(D\right)=-\frac{1}{2}E_{x∼P_{\text{data}}}\left[\log D\left(x\right)\right]-\frac{1}{2}E_{z}\log\left[1-D\left(G\left(z\right)\right)\right]. \end{array}$$

Among them, *G* and *D* represent the differentiable functions of the generator and the discriminator, respectively, *x* is the real data sample, *z* is the random noise vector, and *G*(*z*) is the generated data of the generator. From the perspective of the classifier, the first term of (1) means that *D* is labeled as 1 for real data *x*, and the second term is that *D* is used for generator *G* to map noise *z* into generator-generated data, and *G*(*z*) is labeled as 0. The objective function (1) obtains the optimal solution at:

(2) $$\begin{array}{c} D_{G}^{*}\left(x\right)=\frac{P_{\text{data}}\left(x\right)}{P_{\text{data}}\left(x\right)+P_{g}\left(x\right)}. \end{array}$$

It can be seen from (2) that GAN estimates the ratio of the distribution density of the two concepts, rather than based on the Markov chains or approximating the lower bound of variation. This is the key difference between GAN and other generative models.

From another perspective, the purpose of the discriminator is to correctly distinguish between real data *x* and generated data *G*(*z*), that is, when the input is real data *x*, the output probability value *D*(*x*) should be as close to 1 as possible. When the input is to generate data *G*(*z*) try to make *D*(*G*(*z*)) tend to 0. Combining these two aspects, the formal expression of the objective function of the discriminator is as follows:

(3) $$\begin{array}{c} J\left(D\right)=\max\left\{E_{x∼P_{\text{data}}}\left[\log D\left(x\right)\right]+E_{z}\log\left[1-D\left(G\left(z\right)\right)\right]\right\}. \end{array}$$

Since *G* and *D* play a binary zero-sum game, the objective function of generator *G* is *J*(*G*)=−*J*(*D*). Therefore, the optimization problem of GAN can be described as the following minimax game problem:

(4) $$\begin{array}{c} \underset{G}{\text{min}}\underset{D}{\text{max}}V\left(D,G\right)=E_{x∼P_{\text{data}}}\left[\log D\left(x\right)\right]+E_{z}\log\left[1-D\left(G\left(z\right)\right)\right]. \end{array}$$

Due to the lack of sufficient training in the initial training stage, the data generated by *G* are not realistic enough, so *D* can easily distinguish the generated data from the real data, resulting in insufficient gradient for G. Therefore, training *G* by maximizing log*D*(*G*(*z*)) rather than minimizing log(1 − *D*(*G*(*z*))) is a better strategy.

### 3.3. Objective Function Optimization

#### 3.3.1. Optimal Discriminator

In a continuous space, the mathematical expectation of the objective function (3) can be expanded into the following integral form:

(5) $$\begin{array}{c} V\left(G,D\right)=\int_{x}P_{\text{data}}\left(x\right)\log D\left(x\right)+P_{g}\left(x\right)\log\left(1-D\left(x\right)\right)\text{d}x. \end{array}$$

For any nonzero real numbers a and *b*, and *y* ∈ [0,1], the expression is as follows:

(6) $$\begin{array}{c} a\;\log y+b\;\log\left(1-y\right). \end{array}$$

which takes the maximum value at *a*/*a*+*b*. Therefore, given generator *G*, (5) takes its maximum value at

(7) $$\begin{array}{c} D_{G}^{*}\left(x\right)=\frac{P_{\text{data}}\left(x\right)}{P_{\text{data}}\left(x\right)+P_{g}\left(x\right)}. \end{array}$$

This is the optimal solution of the discriminator *D*. In the actual situation, because the prior *P*_data_(*x*) is not known, the optimal discriminator cannot be obtained by (7). The function of (7) is to prove the existence of the optimal generator *G*. *D*_*G*_^*∗*^ is the value to be approximated by training the discriminator in actual training.

#### 3.3.2. Optimal Generator

The target value of the generator is such that *P*_*g*_(*x*)=*P*_data_(*x*). At this time, the discriminator

(8) $$\begin{array}{c} D_{G}^{*}\left(x\right)=\frac{P_{\text{data}}\left(x\right)}{P_{\text{data}}\left(x\right)+P_{g}\left(x\right)}=\frac{1}{2}. \end{array}$$

That is, it is difficult for the discriminator to distinguish between the generated data and the real data. If and only if *P*_*g*_(*x*)=*P*_data_(*x*), *G* is the global optimal solution of the binary zero-sum game.

### 3.4. Training GAN

Given a generator *G*, D can be obtained by max_*D*_*V*(*D*, *G*). According to (3), the mathematical expectation *E*_*x*∼*P*_data__[log*D*(*x*)] and *E*_*z*_log[1 − *D*(*G*(*z*))] must be obtained. However, the above two expectations cannot be obtained through integration in practice. Therefore, the method of sampling from real data and generated data is used to approximate these two expectations. That is, we take *m* samples from *P*_data_(*x*){*x*_1_, *x*_2_,…, *x*_3_, } and take *m* samples from $P_{g}\left(x\right)\left\{\tilde{x_{1}},\tilde{x_{2}},\ldots ,\tilde{x_{3}}\right\}$.

(9) $$\begin{array}{c} \text{max}V\left(D,G\right)\approx \max\tilde{V}=\frac{1}{m}\sum_{i=1}^{m}\log D\left(x^{i}\right)+\frac{1}{m}\sum_{i=1}^{m}\log D\left({\tilde{x}}^{i}\right). \end{array}$$

**Figure 3:**
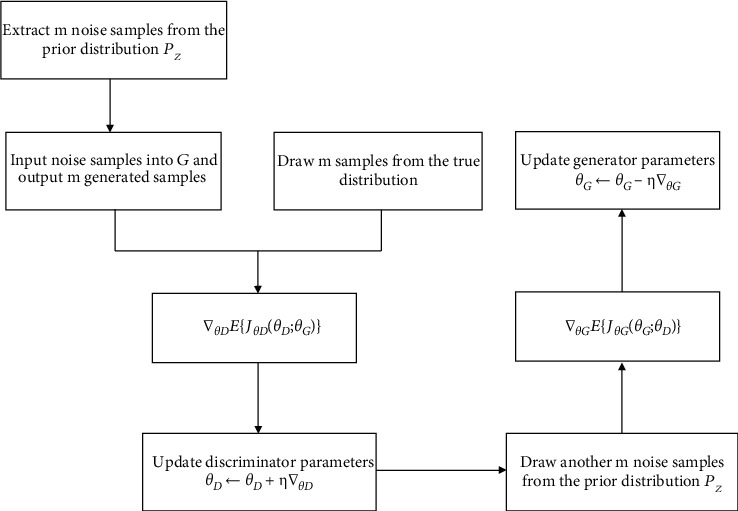
Parameter update flowchart.

Each round of the parameter update process is shown in Figure 3:

The challenge of how to balance generator *G* and discriminator *D* is a very important issue. In actual training, in the same round of parameter update, the parameters of each pair of discriminator *D* are updated *k* times, and the parameters of generator *G* are updated once. Otherwise, generator *G* will easily collapse to the saddle point.

**Figure 4:**
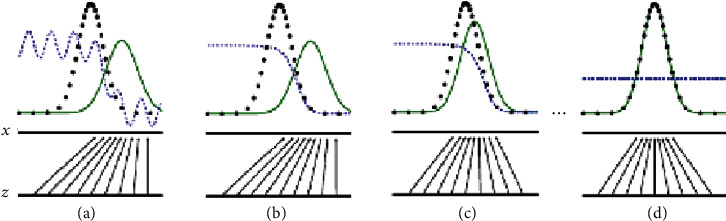
Schematic diagram of GAN's minimax game training process.

Figure 4 depicts this process intuitively. The equidistant horizontal lines indicate that the data in the sampling domain are uniformly distributed. The arrow pointing from *z* to *x* indicates that the random noise vector *z* is mapped to generated data by *x*=*G*(*z*). In Figure 4(a), the probability distribution of the generator *P*_*g*_(*x*) (green line) has some differences between the probability distribution *P*_data_(*x*) (black line) of the real data, discriminator output value (blue line) is higher on the left and lower on the right, which means that the discriminator can still accurately distinguish true and false data in the current state (*D*(*x*) tend to 1 and *D*(*G*(*x*)) tend to 0). As the number of training increases, Figures 4(b) and 4(c) show the process of the generated distribution gradually approaching the true distribution: In Figure 4(b), fix *G* and train *D* to converge to the optimal solution *D*^*∗*^=(*P*_data_/*P*_data_+*P*_*g*_); 4(c) Fix *D*. After *G* is updated, the gradient of *D* moves *G* to the area where *D* will make a wrong judgment. If both *G* and *D* have very strong learning ability, they will eventually reach *P*_*g*_=*P*_data_, that is, the generated distribution is completely consistent with the real distribution, as shown in Figure 4(d). At this time, the state of *D* cannot distinguish between real data and generated data, that is *D*=1/2, the Nash equilibrium is reached.

## 4. Case Study Based on GAN

### 4.1. Rolling Bearing Data Description

First, we process the actual rolling bearing data, build a complete GAN network and train, compare the generated data of the normal state, moderate degradation state, and rapid failure state of the bearing with the real data, and verify the similarity of the generated data to prove the feasibility of the method.

**Figure 5:**
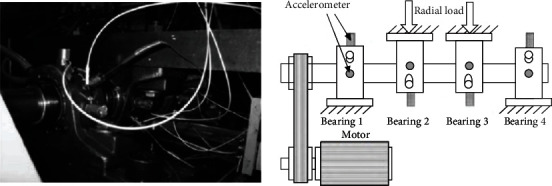
Cincinnati Lab.

This paper uses bearing data from the University of Cincinnati, and the test bench is shown in Figure 5. The bearing life test bench of the University of Cincinnati is to install four bearings on the shaft, drive the main shaft connected to the shaft through the transmission of the belt, and keep the shaft speed constant at 2000 rpm. Apply a radial load of 6000 pounds to the shaft and bearings through a spring mechanism. At the same time, all bearings are forced to be lubricated.

The experimental bearing model is Rexnord ZA-2115 double row bearing. The structural parameters of the bearing are shown in Table 1. As shown in Figure 5 above, a PCB353B33 high-sensitivity quartz ICP accelerometer is mounted on the bearing box. Each bearing is equipped with two horizontal (*x* direction) and vertical (*y* direction) accelerometers. The vibration signals collected by the sensors constitute data set 1, 2 and 3. All failures occurred after 100 million revolutions in the design life, that is, all failures occurred after the design life was exceeded. The running time of the collected data was from 10:32:39 on February 12, 2004 to 06:22:39 on February 19, 2004, and finally stopped due to a failure of the outer ring of the bearing 1.

According to the bearing structure parameters shown in Table 1 and the calculation formula of the fault characteristic frequency given in Chapter 3, the fault characteristic frequency of the test bearing can be obtained, as shown in Table 2.

**Figure 6:**
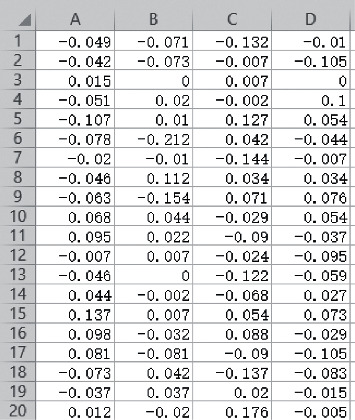
Partial real data samples.

Considering the training time and other factors, the data period of data set 2 is relatively short, so this article uses data set 2 as the learning data of GAN. This set of data has a total of 984 files, and each file has 20480 data. The specific description of the data is as follows as shown in Table 3. The 4 acquisition channels correspond to *A*, *B*, *C*, and *D*, where *A* corresponds to the information collected by bearing 1, and so on, corresponding to the collected information of 4 bearings. This article uses three stages of data as learning data, the 1 to 702 files (normal bearing state), the 703 to 968 files (moderate bearing degradation), and the 969 to 984 files (fast bearing failure). Partial bearing data are shown in Figure 6.

**Figure 7:**
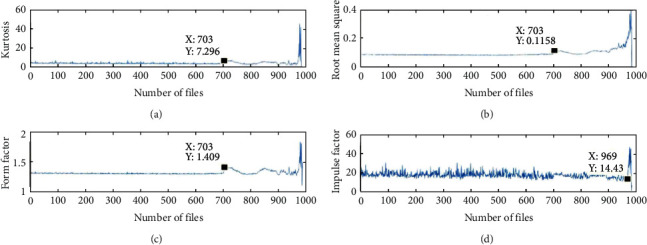
Time domain characteristic change diagram during the life cycle of the bearing.

According to the time-domain characteristic parameter expressions in Chapter 3, the change curves of the four time-domain characteristic indexes of the bearing life cycle kurtosis value, root mean square value, form factor, and pulse factor are calculated, as shown in Figure 7.

By observing the change curve of the four time-domain characteristic indicators shown in Figure 7 during the full life cycle of the bearing, we can know the following:

The amplitude shown in Figures 7(a)–7(c) shows a certain jump near 703 documents (about 117 h), and there are long-term fluctuations, indicating that the azimuth time at this position has shown “moderate degradation.” Since then, the trend of the RMS value is consistent with the trend of the failure development, and the kurtosis value fluctuates greatly in the later stage of the fault development, which cannot accurately reflect the changing law of the bearing health status.

Figures 7(a)–7(d). After 969 documents (about 161 h), the amplitude suddenly soars, which indicates that the bearing has been severely degraded and is in a state of “rapid failure.”

In summary, the training data are divided into 3 parts to prepare for the following training. The first part (files 1 to 702) is the data under normal bearing conditions and the second part (files 703 to 969) is the data of the moderately degraded state of the bearing. The third part (files 969 to 984) is the rapid failure state of the bearing, that is, the failure state.

### 4.2. The GAN Network Construction

#### 4.2.1. Network Structure

**Figure 8:**
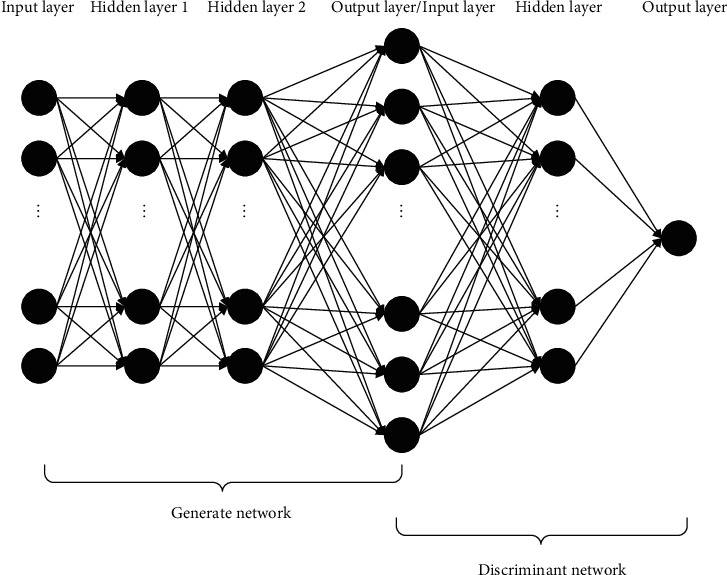
GAN structure diagram.

A complete GAN is composed of two parts, one is the generative model and the other is the discriminative model. The generator model has 4 layers including an input layer, two hidden layers, and an output layer. The discriminant model is a three-layer neural network, including an input layer, a hidden layer, and an output layer. The two neural networks are connected by the last layer of the generator and the first layer of the discriminator to form a generative adversarial network, as shown in Figure 8.

#### 4.2.2. GAN Network Model Input and Output

In combination with the test requirements, the experimental data in this article are the full life data of Cincinnati rolling bearings. A single sample is taken for a period of time, and a single sample is a 1 × 20480 vector. At the same time, the output result of the generator is also a 1 × 20480 vector.

In the fault diagnosis of the bearing, the fault condition of the state is mapped from the real state. Therefore, from the perspective of mechanism analysis and experimental results, the input layer of the generated network is a 1 × 128 noise signal, the hidden layer 1 and the hidden layer 2 have 128 neurons, and the output layer has 20480 neurons. The input layer is fully connected with the hidden layer and the output layer.

The input layer of the discriminant network is the output layer of the generating network, that is, 20480 neurons, the hidden layer has 128 neurons, and the output layer has only one neuron. The input layer is fully connected with the hidden layer and the output layer.

#### 4.2.3. Model Hyperparameters

The hyperparameters in the GAN model mainly include the number of iterations of model structure parameters, etc. The values of these parameters will directly affect the final generation result.

1. Structural parameters
2. The generator uses a 4-layer neural network, and the discriminator uses a 3-layer network structure. In the original GAN, the image signal is mapped from the noise, but in the fault diagnosis, the image signal is mapped from a real normal working condition. The fault signal under the same working condition. Therefore, considering the intuitive mechanism and experimental results, the generator uses a 4-layer network. At the same time, the number of neurons in the input layer and the hidden layer does not increase layer by layer, that is, the number of neurons in each layer is the same, and the number of neurons in each layer is 128. The discriminant network adopts a three-layer structure. The first layer coincides with the last layer of the generator. The second reduces the number of neurons to 128. The output layer of the last layer is only one neuron because it only needs the probability of outputting data.
3. Transfer function uses the rule function, and at the same time, to match the transfer function, the learning rate of the generating network and the discriminant network is a small 0.0001. The last layer of the discriminant network only needs to output a probability of 0–1, so the sigma function is adopted.

### 4.3. The Bearing Game Generated Data Training Based on GAN

In this section, we program the GAN through Python, the simulation data are generated and compared with the real data under the normal state, the moderately degraded state, and the rapid failure state of the bearing.

#### 4.3.1. GAN-Based Training under the Normal State

This training uses the normal bearing state data in the 1st file to the 702nd file and compares them with the real data to verify the feasibility of the generated data. The analysis in this section is the comparison between the generated data and the real data in the same time period.

**Figure 9:**
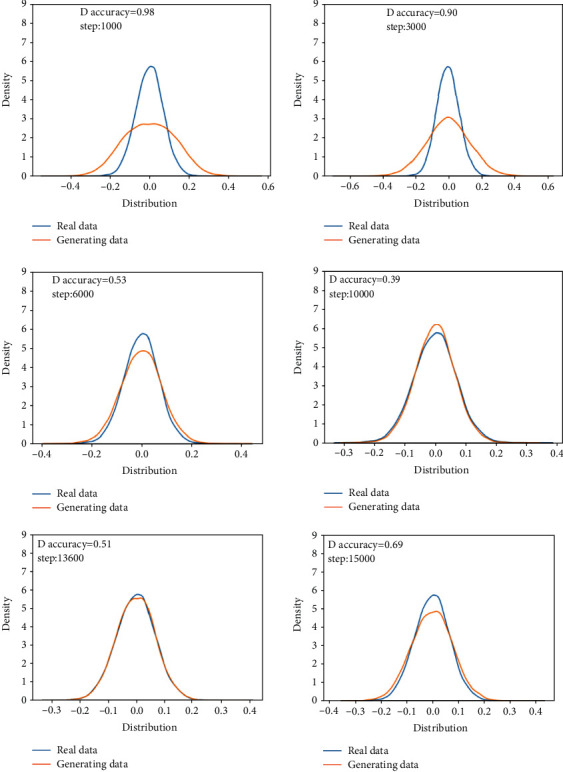
Comparison of the probability distribution of real data and generated data under the normal state.

**Figure 10:**
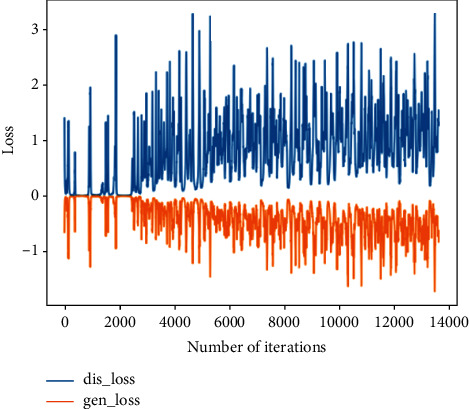
Generating network and discriminating network loss function in the normal state.

**Figure 11:**
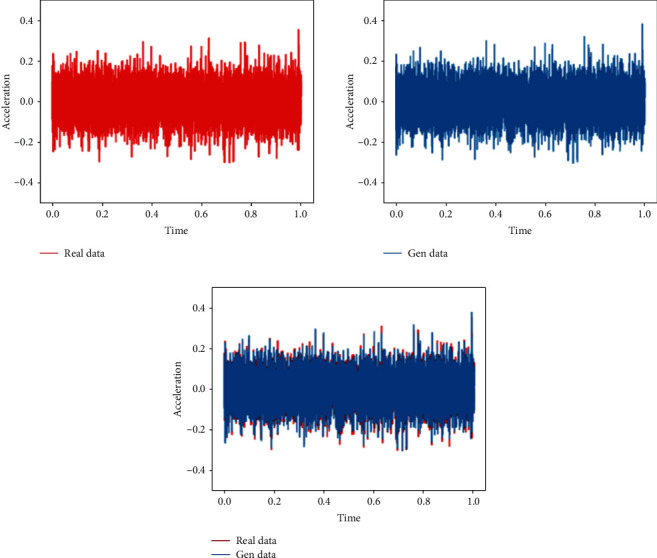
Time-domain diagram of generated data and real data under the normal state.

**Figure 12:**
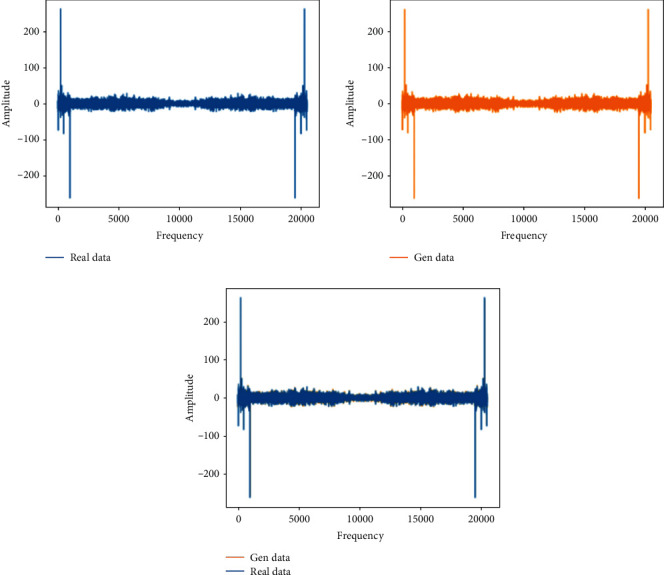
Frequency domain distribution of real data and generated data under the normal state.

1. Probability distributions
2. When setting the training termination condition, we gradually expand the number of training rounds from small to large until the probability distribution is close to the real data. The training process is shown in Figures 9, 9(a)∼9(f) are, respectively, selected as the comparison results of the probability distribution of 1000, 3000, 6000, 10000, 13600, and 15000 rounds.
3. After experiments, it is found that under the above parameters and experimental data, the number of rounds can achieve better results at about 13,600 times. At this time, the discriminant score of the discriminant network is 0.51.
4. In the process of training the normal data, the loss function of the generating network and the discriminating network change as shown in Figure 10. It can be seen that the generative network and the discriminant network are fighting against each other, and neither side wants the other to win.
5. Time-domain characteristics
6. Through the comparison of the 6 indicators of mean, root mean square, skewness, kurtosis, form factor, maximum, and minimum in Table 4, it is found that the real data and the generated data under normal bearing conditions are very similar. The maximum relative deviation of the mean is 6.2%, and the minimum relative deviation is 0.
7. Time-domain distribution
8. It can be seen from the time-domain waveform diagram in Figure 11 that the generated data and the real data are very similar at different times, but they are not the same data.
9. Frequency domain distribution
10. The generated data and the real data are subjected to the Hilbert transform and Fourier transform to obtain the frequency domain waveform diagram, as shown in Figure 12. It can be seen that the frequency domain waveforms of the real data and the generated data are very similar. In summary, the generated data under normal conditions can be used as experimental research data.

#### 4.3.2. Training under the Moderate Degradation State

This training uses the moderately degraded bearing state data in 703rd file to the 968th file and compares them with the real data to verify the feasibility of the generated data. The analysis in this section is the comparison between the generated data and the real data in the same time period.

**Figure 13:**
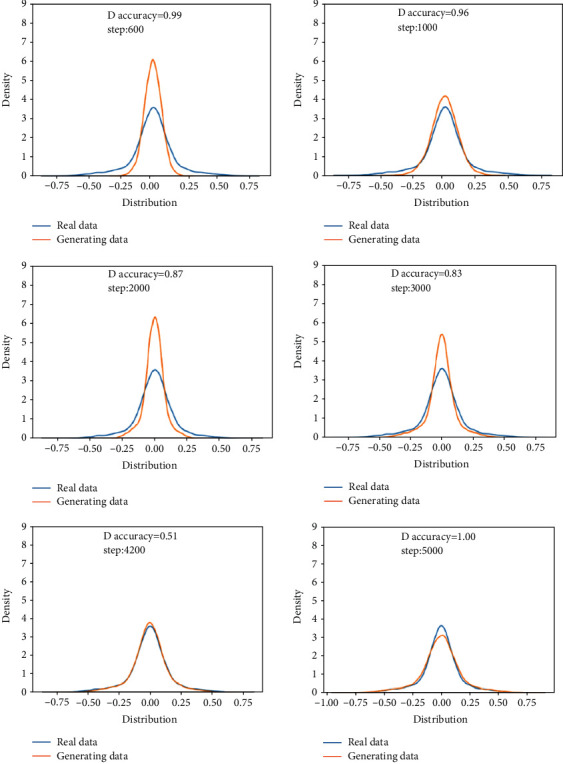
Comparison of the probability distribution of real data and generated data under the moderate degradation state.

**Figure 14:**
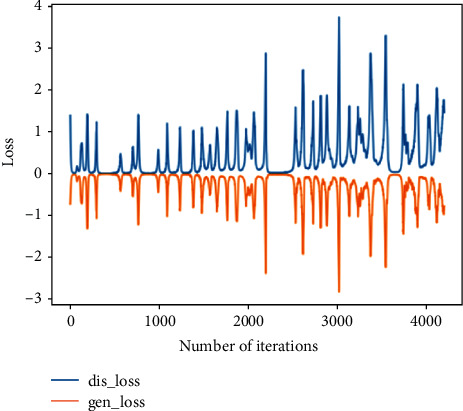
Loss function of generating network and discriminating network in bearing the moderate degradation state.

**Figure 15:**
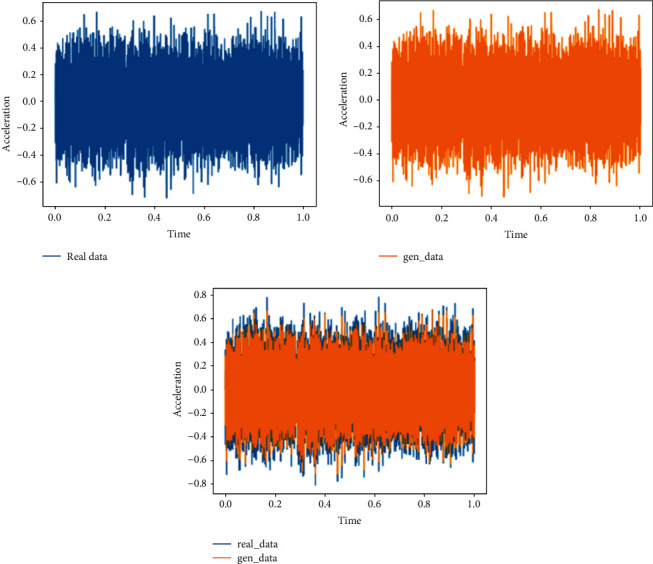
Time-domain diagram of generated data and real data under the moderate degradation state.

**Figure 16:**
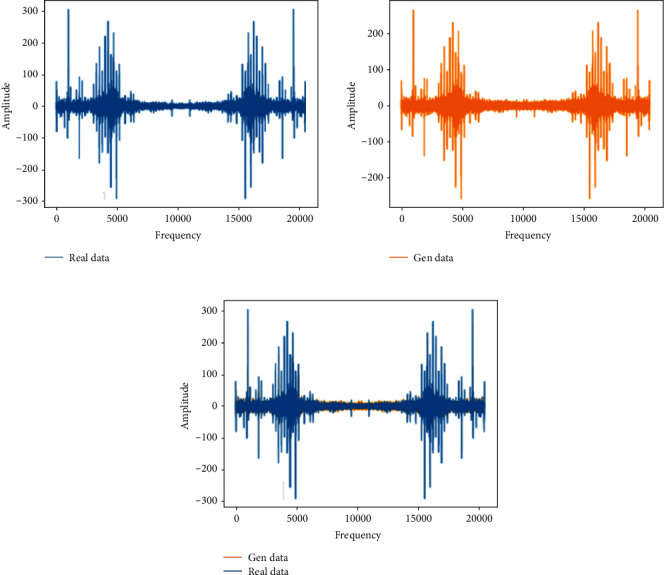
Frequency domain distribution of real data and generated data under the moderate degradation state.

1. Probability distributions
2. When setting the training termination condition, we gradually expand the number of training rounds from small to large until the probability distribution is close to the real data. The training process is shown in Figures 13, 13(a)∼13(f) are, respectively, selected as the comparison results of the probability distribution of 600, 1000, 2000, 3000, 4200, and 5000 rounds.
3. After experiments, it is found that under the above parameters and experimental data, the number of rounds can achieve good results at about 4200 times. At this time, the discriminant score of the discriminant network is 0.51.
4. In the process of training the bearing's moderately degraded data, the loss function of the generating network and the discriminating network change as shown in Figure 14. It can be seen that the generating network and the discriminating network are fighting against each other, and neither party wants the other to win.
5. Time-domain characteristics
6. Through the comparison of the 6 indicators of mean, root mean square, skewness, kurtosis, form factor, maximum, and minimum in Table 5, it is found that the real data and the generated data under the condition of moderate bearing degradation are very similar, and the maximum relative deviation of the maximum value is 6.2%, the minimum relative deviation of the form factor is 1.1%.
7. Time-domain distribution
8. It can be seen from the time-domain waveform diagram in Figure 15 that the generated data and the real data are very similar at different moments, but they are not the same data.
9. Frequency domain distribution
10. The generated data and the real data are subjected to the Hilbert transform and Fourier transform to obtain the frequency domain waveform diagram, as shown in Figure 16. It can be seen that the frequency domain waveforms of the real data and the generated data are very similar. In summary, the generated data under moderate degradation conditions can be used as experimental research data.

#### 4.3.3. Training under the Rapid Failure State

This training uses the rapid failure state data for training in 969th file to the 984th file and compares them with the real data to verify the feasibility of the generated data. During training, it is found that the discriminant network can always distinguish the true and false of the data, so the generator is strengthened, and the structure of one input layer, four hidden layers and one output layer is used for training. The analysis in this section is the comparison between the generated data and the real data in the same time period.

**Figure 17:**
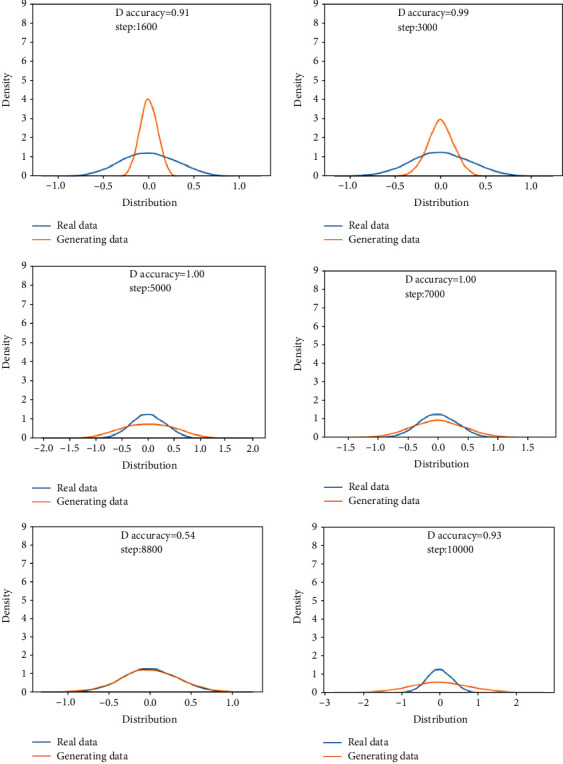
Comparison of the probability distribution of real data and generated data under the rapid failure state.

**Figure 18:**
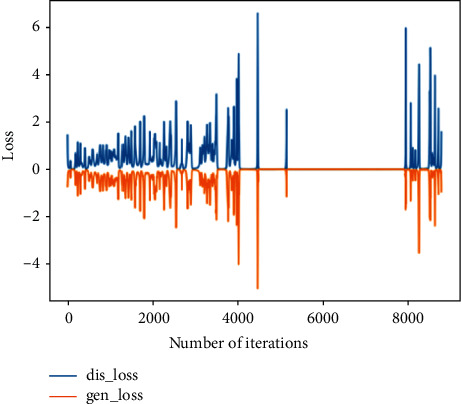
Generating network and discriminating network loss function in the rapid failure state.

**Figure 19:**
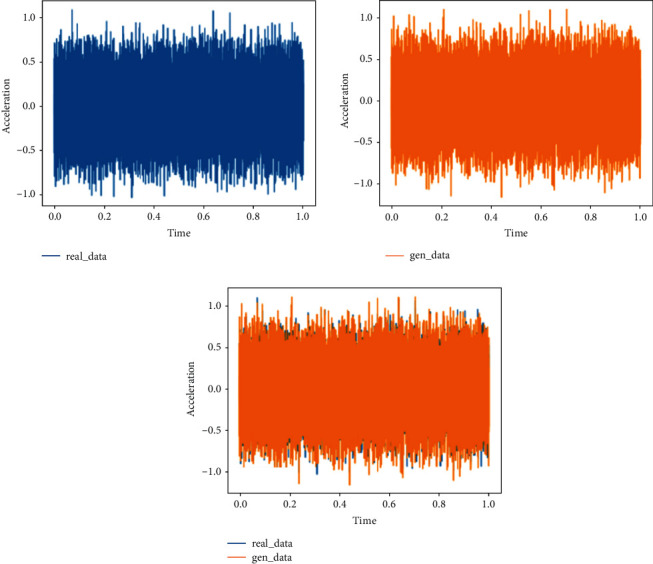
Time-domain diagram of generated data and real data under the rapid failure state.

**Figure 20:**
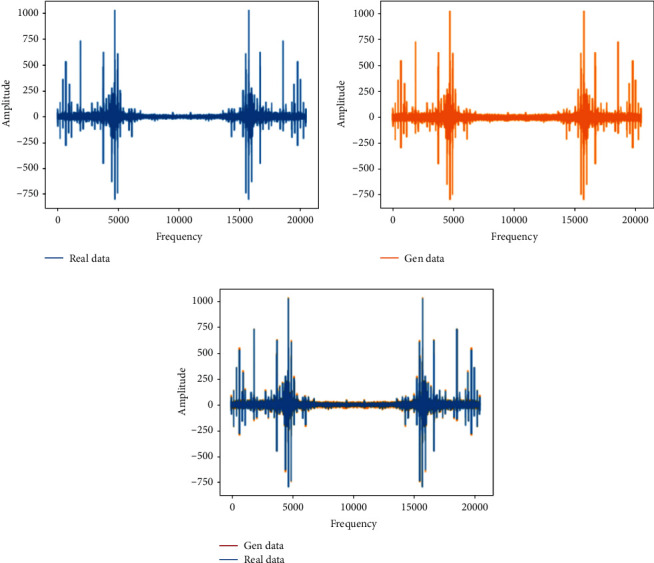
Frequency domain distribution of real data and generated data under the rapid failure state.

1. Probability distributions
2. When setting the training termination condition, gradually expand the number of training rounds from small to large until the probability distribution is close to the real data. The training process is shown in Figure 17. Figures 17(a)∼17(f) are selected as the comparison results of the probability distribution of 1600, 3000, 5000, 7000, 8800, and 10000 rounds, respectively.
3. After experiments, it is found that under the above parameters and experimental data, the number of rounds can achieve good results at about 8800 times. At this time, the discriminant score of the discriminant network is 0.54.
4. In the process of training the bearing's rapid failure data, the loss functions of the generating network and the discriminating network change as shown in Figure 18. It can be seen that the generating network and the discriminating network are fighting against each other, and neither party wants the other to win. When the confrontation reached between 4500 and 7800 times, the two sides reached a balance, and then confronted again.
5. Time-domain characteristics
6. Through the comparison of 6 indicators of mean, root mean square, skewness, kurtosis, form factor, maximum, and minimum in Table 6, it is found that the real data and the generated data under the rapid bearing failure state are very similar, and the relative deviation of the form factor is maximum 0.1%, the minimum relative deviation of the mean is 17%.
7. Time-domain distribution
8. It can be seen from the time-domain waveform diagram in Figure 19 that the generated data and the real data are very similar at different times, but they are not the same data.
9. Frequency domain distribution
10. The generated data and the real data are subjected to the Hilbert transform and Fourier transform to obtain the frequency domain waveform diagram, as shown in Figure 20. It can be seen that the frequency domain waveforms of the real data and the generated data are very similar. In summary, the generated data under rapid failure conditions can be used as experimental research data.

By training the bearing data in the three states and comparing it with the real data, it is found that the generated data and the real data are very similar to the real data in both the time domain and the frequency domain and can replace the real data for experimental research. When generating the bearing operation data in the normal state, the number of training times is 13600 times, the number of training times in the moderately degraded state is at least 4200 times, and the number of the rapid failure state (fault state) is 8800 times. At the same time, the generation network structure of the normal state and the moderately degraded state is 4 layers, and the number of generation network layers in the fast failure state reaches 6 layers.

## 5. Conclusion

This paper proposes a new way to generate bearing data based on the generative adversarial network method in order to solve fault diagnosis problems with insufficient data. Through the time-domain characteristic analysis of the rolling bearings life cycle monitoring data in actual operation, the actual bearing data are divided into three periods: normal state, moderate degradation state, and rapid failure state. The GAN-based generated network model is then studied for the construction and training. Comparing the generated data with the real data in the time domain and the frequency domain, respectively, and verifying the similarity between the generated data and the real data, the game generated data method shows its effectiveness and that it can provide new enlightenment for solving the insufficient data problem in the fault diagnosis field. At last, the pseudoreal data game generated by GAN model is verified highly similar to the real data and showed the possibility of its application in the fault diagnosis field.

In future work, the universality of the new data set will be further verified to ensure that various data sets can be successfully applied. Moreover, the more fault diagnosis model could be built using multi-category generating bearing data to verify the diagnosis effect.

## Figures and Tables

**Table 1:** Rexnord ZA-2115 bearing parameters.

Bearing pitch diameter (mm)	Rolling element diameter (mm)	Number of balls (each column)	Rolling element contact (°)
*D* = 71.5	*D* = 8.4	*Z* = 16	*α* = 15.17

**Table 2:** Fault characteristic frequency.

Outer ring (Hz)	Inner circle (Hz)	Rolling element (Hz)	Cage (Hz)
236.4	296.9	140.0	14.8

**Table 3:** Data set description information.

Collection time	From 10:32:39, February 12, 2004, to 06:22:39, February 19, 2004
Number of files	984
Number of channels	4
Channel settings	Bearing 1-channel 1; bearing 2-channel 2; bearing 3-channel 3; bearing 4-channel 4;
Collection interval	10 minutes
Vibration signal length	20480 dots (each bearing)
File format	ASCII
Test results	Failure of bearing 1 outer ring

**Table 4:** Time-domain characteristics of real data and generated data under the normal state.

	Real data	Generate data	Relative deviation(%)
Mean	−0.0017	−0.0015	6.2
Root mean square	0.0701	0.0699	0.1
Skewness	−0.0302	−0.0205	19
Kurtosis	0.3393	0.2741	11
Form factor	1.2657	1.2589	0.3
Maximum	0.354	0.378	3.2
Minimum	−0.3	−0.3	0

**Table 5:** Time-domain characteristics of real data and generated data under the moderate degradation state.

	Real data	Generate data	Relative deviation(%)
Mean	−0.0019	−0.0017	5.6
Root mean square	0.1669	0.1486	5.8
Skewness	−0.1350	−0.1202	5.8
Kurtosis	2.3899	2.0825	6.9
Form factor	1.4328	1.4029	1.1
Maximum	0.776	0.67	7.3
Minimum	−0.808	−0.717	6

**Table 6:** Time-domain characteristics of real data and generated data in the rapid failure state.

	Real data	Generate data	Relative deviation(%)
Mean	−0.0019	−0.0027	17
Root mean square	0.3148	0.3294	2.3
Skewness	0.0079	0.0075	2.6
Kurtosis	−0.2586	−0.2075	11
Form factor	1.2397	1.2421	0.1
Maximum	1.086	1.096	0.5
Minimum	−1.025	−1.155	6

## Data Availability

The data used to support the findings of this study are available from the corresponding author upon request.
